# Tailoring support following summative assessments: a latent profile analysis of student outcomes across five medical specialities

**DOI:** 10.1007/s10459-024-10357-9

**Published:** 2024-07-23

**Authors:** Huiming Ding, Matt Homer

**Affiliations:** School of Medicine, University of Leeds, Leeds, UK

**Keywords:** Latent profile analysis, Summative assessment, AKT, OSCE, Specialities, Feedback

## Abstract

Summative assessments are often underused for feedback, despite them being rich with data of students’ applied knowledge and clinical and professional skills. To better inform teaching and student support, this study aims to gain insights from summative assessments through profiling students’ performance patterns and identify those students missing the basic knowledge and skills in medical specialities essential for their future career. We use Latent Profile Analysis to classify a senior undergraduate year group (*n* = 295) based on their performance in applied knowledge test (AKT) and OSCE, in which items and stations are pre-classified across five specialities (e.g. Acute and Critical Care, Paediatrics,…). Four distinct groups of students with increasing average performance levels in the AKT, and three such groups in the OSCE are identified. Overall, these two classifications are positively correlated. However, some students do well in one assessment format but not in the other. Importantly, in both the AKT and the OSCE there is a mixed group containing students who have met the required standard to pass, and those who have not. This suggests that a conception of a borderline group at the exam-level can be overly simplistic. There is little literature relating AKT and OSCE performance in this way, and the paper discusses how our analysis gives placement tutors key insights into providing tailored support for distinct student groups needing remediation. It also gives additional information to assessment writers about the performance and difficulty of their assessment items/stations, and to wider faculty about student overall performance and across specialities.

## Introduction

Summative assessment like Applied Knowledge Tests (AKTs) and Objective Structured Clinical Examinations (OSCEs), provide a key opportunity for generating appropriate feedback for students and faculty to facilitate the improvement of learning and curriculum and aid the development of the academic/clinical programme (Lineberry, 2020; Norcini et al., 2018; Park et al., 2020). However, summative data are often underused in this respect, despite the well-known calls to promote the formative use of summative assessment outcomes (Black et al., 2003; Lineberry, 2020; Schuwirth & van der Vleuten, 2011). In practice, focus is often put on the pass-fail decision which is although certainly important (Harrison et al., 2015) with assessment data commonly aggregated into single scores regarding overall performance, and perhaps also performance in different domains or speciality areas (e.g. Acute and Critical Care, Psychiatry,…) for use in student feedback. It is not always clear to what extent students meaningfully engage with such feedback to develop their knowledge and improve their clinical skills (Hattie & Timperley, 2007). As summative assessments are usually administered at the end of the academic year, their primary function is often considered by students as evaluation for progression rather than informing learning and development. Feedback after these types of assessments is often under-used by students, especially for those who have passed the exam (Harrison et al., 2013, 2015, 2016) despite their acknowledged compensatory nature where overall score might mask weak performance in some areas (Cizek & Bunch, 2007). Medical schools, with limited faculty resources, naturally tend to focus their feedback efforts on those who failed the exam (Harrison et al., 2013). This implies that passing students might not always have an accurate conception of their competence, particularly in sub-domains. Further, evidence suggests that low performers are those who are more likely to struggle to set appropriate learning goals to address specific weak areas (Chang et al., 2011). This suggests that schools should better identify the students in need of support in specific areas, provide sufficient learning opportunities, and develop students accordingly (GMC, 2015).

It is therefore important to identify and understand the performance patterns of distinct sub-groups of students so that tailored remediation can be implemented to effectively address the specific weaknesses of such sub-groups, thereby improving future learning. Nevertheless, single scores per se do not directly indicate specific patterns of knowledge and skills for sub-groups (Altshuler et al., 2023). By contrast, the person-centred methodological approach, latent profile analysis (LPA) (or latent class analysis, LCA)^1^, assumes that groupings of individuals, which are not directly observable in the data, can be revealed *a posteriori* based on the analysis of observed scores or responses (Jung & Wickrama, 2008; Marsh et al., 2009). This approach identifies the underlying latent heterogeneity in students (Lubke & Muthén, 2005) and classifies them into distinct sub-groups who have shared performance characteristics (Weller et al., 2020) based on the set of measures (scores or sub-scores) used. Compared with a simple pass/fail classification, or aggregate scores or rankings, LPA allows for the identification and understanding of the particular needs of sub-groups of students according to their common characteristics (i.e. relative strengths and weakness). It also provides additional insights that can help deliver adaptive teaching (Boscardin, 2012; Ma, 2021) based on scoring patterns identified in the summative examinations.

In terms of the limited LPA/LCA literature in medical education, Boscardin (2012) profiled students’ history taking, physical exam, and physician-patient interaction skills in the Clinical Performance Examination (CPX). Besides identifying two different groups of students for remediation – one group needs support for all the three types of clinical skills across the related scenario cases while the other group needs support particularly for physician-patient interaction skills, her study also suggests that setting a cut-off value for each CPX case instead of an overall cut score is preferred to ensure all the ‘pass’ students are those having the necessary skills in all the clinical areas (Boscardin, 2012). More recently, she and colleagues (2022) used LPA-type methods to explore student profiles of learning orientation, learning approaches, and perception of the learning environment. In their study, the four identified profile groups were found related to students’ clerkship performance (Boscardin et al., 2022). In another LPA study identifying the latent profiles of medical students’ four domains of communication skills demonstrated in the Comprehensive Clinical Skills Exam (CCSE), Altshuler et al. (2023) evidenced the typical weaknesses of the low performing groups which led to them failing the exam. Their findings provide detailed guidance on the provision of targeted feedback and personalised education on communication skills. Mak-van der Vossen et al. (2016) identified three different profiles of medical students’ unprofessional behaviour, which were labelled as (1) “Poor reliability”, (2) “Poor reliability and poor insight”, and (3) “Poor reliability, poor insight and poor adaptability”. LPA has also been used to sort different specialties into distinct groups sharing similar intrinsic characteristics in terms of working environment (e.g. research intensive, procedurally-focused, clinical focused, etc.), which can then be used to inform medical students on their specialty choice (Balas et al., 2023).

Acquisition of basic clinical knowledge and skills across different clinical specialties is essential for medical students to prepare themselves for undertaking medical practice as doctors in various healthcare settings and lays the foundation for their future career choices (GMC, 2015, 2022; VanOrder et al., 2017). To our knowledge, few studies have investigated whether there are distinct patterns of students’ performance across different clinical specialities, and how those patterns differentiate across distinct groups of students in both applied knowledge tests and OSCE-type assessments simultaneously. According to Zelesniack et al. (2022), advanced undergraduate medical students lack proper understanding of specific competencies that are required for different specialties. Some researchers have also suggested that “acute care is an area of concern for UK graduates” in medicine (Tallentire et al., 2012, p.365) due to the lack of exposure and hands-on experience in medical school (Burridge et al., 2020). Nevertheless, these claims were made based on doctors’ perceptions rather than real assessment data.

At the test level, contradictory results have been reported on the correlation between OSCE scores and knowledge test scores in some extant studies (e.g. Auewarakul et al., 2005; Johnson & Reynard, 1994; Nguyen et al., 2023) suggesting that different assessment formats do measure different constructs/domains, at least in some contexts. Students’ performance in an OSCE may also be influenced by factors such as their communication skills (Attrill et al., 2016; Groene et al., 2022), examiner effects (e.g. Chong et al., 2017; Homer, 2023), and testing context (Blaskiewicz et al., 2004). However, there is scarce research discussing the extent to which profiles of what students “know” and “know how” in the knowledge test align with what they can actually “show how” in practice (i.e. clinical and other skills as measured in the OSCE) (Miller, 1990). More nuanced research is therefore needed that links both AKT and OSCE data, holistically looking into areas of student relative strength and weakness in applied knowledge and clinical skills.

In this study, to better inform teaching and tailor student support, we gain useful insights from AKT and OSCE assessment data at the speciality level by using LPA as our main methodological approach. Our research questions are the following.

1. How do students group according to AKT outcomes at speciality level?
2. How do students group according to OSCE outcomes at speciality level?
3. How well do these groupings align with each other?
4. What insights can all stakeholders gain from this type of profiling?

## Methods

### Data and sample

The datasets that we employed in this study consist of the Undergraduate medicine MBChB programme Year 4 students’ summative assessment data from the AKT and the OSCE collected at the end of a recent post-covid academic year at University of Leeds, UK. Both assessments are part 1 of a sequential test (Cookson et al., 2011; Muijtjens et al., 2000; Pell et al., 2013). According to UK’s General Medical Council (GMC), “medical school curricula must give medical students:… experience in a range of specialties, in different settings, with the diversity of patient groups that they would see when working as a doctor” (GMC, 2015, p.33). In Year 4, learning focuses on developing clinical experience through the five Integrated Course Units (ICUs) of speciality placements in Acute and Critical Care (ACC), Continuing Care and Cancer (CCC), Gynaecology, Obstetrics and Sexual Health (GOSH), Paediatrics, and Psychiatry. Each ICU takes six weeks. In addition, three days of Integrated “Theme” teaching across the year provide students with opportunities to integrate their knowledge across different specialities. By the end of the academic year, students are required to have acquired all mandatory skills of these specialities, which prepare them to be a safe foundation year doctor, build a basis for their future career choices, and enhance their capabilities of working effectively in interprofessional teams. Students’ knowledge and skills in the same five specialities are assessed across both the summative AKT and OSCE. The size of the Year 4 cohort is 295. The University of Leeds gave permission for this anonymized data to be used for research. The Chair of the University of Leeds School of Medicine research ethics committee and the Head of Assessment at the School confirmed to the authors that formal ethics approval for this study was not required as it involved the use of routinely collected student assessment data which were fully anonymized prior to analysis.

The AKT (Cronbach’s alpha = 0.82) consisted of 147 multiple-choice items covering the five specialities with around 30 item each, all scored 0/1. The OSCE (omega.total = 0.78) consisted of 14 stations, assessing students’ clinical skills relevant to the five specialities (and also communication skills and other generic skills). Each specialty was the focus in two stations, and the other four stations had more integrated scenarios in terms of covering content tasks across the specialities. Students’ percent correct scores in specialities in the AKT and in the OSCE were calculated and used as the indicator variables for LPA modelling. Five such indicators by speciality were constructed based on the AKT dataset. Similarly, five indicators, one for each speciality, and one indicator for integrated specialities, were created based on the OSCE checklist scores provided by examiners.

Table 1 summarises the descriptive statistics of students’ performance for each speciality indicator. It also includes the speciality-level ‘percent pass marks’ based on the relevant Ebel ratings (McKinley & Norcini, 2014) for AKT, and borderline regression (Wood et al., 2006) for OSCE.

**Table 1:** Descriptive statistics of the AKT and OSCE, by specialities

	Variables	N of items/stations	% pass marks	% correct scores			
				Mean	SD	Min	Max
AKT	AKT_ACC	30	54.70	60.24	11.94	23.33	90.00
	AKT_CCC	30	50.93	61.41	9.50	26.67	86.67
	AKT_GOSH	28	53.11	73.69	9.77	42.86	96.43
	AKT_Paediatrics	30	46.60	64.33	10.76	26.67	86.67
	AKT_Psychiatry	29	44.38	72.45	10.39	31.03	93.10
OSCE	OSCE_ACC	2	39.29	49.33	13.71	14.29	80.36
	OSCE_CCC	2	53.85	60.61	11.41	16.07	87.50
	OSCE_GOSH	2	51.79	61.54	11.24	25.00	91.07
	OSCE_Paediatrics	2	30.91	45.28	15.01	10.91	85.45
	OSCE_Psychiatry	2	50.00	64.32	11.42	31.03	91.38
	OSCE_ICUint	4	51.89	63.41	9.47	35.85	87.74

### Data analyses

#### Latent profile analysis

Data analyses were conducted in Stata 18 (StataCorp, 2023). We first ran latent profile analysis to identify different groups of students, using students’ percent correct scores on the speciality variables. This was done separately for the AKT and OSCE data. There are various fit indices to evaluate the goodness of fit of LPA model solutions statistically and inform a decision on the number of profiles (i.e. student groups). The indices Akaike’s information criterion (AIC) (Akaike, 1974) and Schwarz’s Bayesian information criterion (BIC) (Schwarz, 1978), which are available in Stata 18, were employed in this study. Lower values of AIC and BIC indicate better model fit. BIC is also considered by some researchers as the most reliable indicator (Nylund et al., 2018; Vermunt, 2002) and indicates parsimony as well (Weller et al., 2020). In the case where the lowest BIC and the lowest AIC are observed in different models, the “elbow points” of the changes of these indicator values can be considered to determine the optimal model (Nylund-Gibson & Choi, 2018; Petras & Masyn, 2010).

With the one-profile solution as the default, we conducted two–profile to six–profile solutions based on the AKT data and the OSCE data respectively, and then identified the optimal model for each dataset. We also examined entropy (Medeiros, 2022) and the average posterior probability to evaluate the accuracy of profile classification. Entropy values range from 0 to 1, with higher values indicating stronger classification certainty (Jung & Wickrama, 2008). It is suggested that an entropy value above 0.80 is preferred, and a value between 0.60 and 0.80 is considered acceptable (Muthén, 2004; Weller et al., 2020). The average posterior probabilities indicate the average probability at which students are accurately classified into a profile (Muthén & Muthén, 2000). As suggested by Weller et al. (2020), a cut-off value greater than 0.80 is acceptable and higher than 0.90 is ideal for ensuring classification accuracy. Additionally, group size was also considered, following the suggestion that the profile with less than 1% of the student sample should be rejected (Spurk et al., 2020).

#### Comparing profiles with ANOVA

With the profiles determined based on the statistical information criteria, conceptual interpretability and classification size, we used analysis of variance (ANOVA) to compare the mean AKT performance of students of different profile groups in the specialities, and compared these profiles with the overall pass/fail groups as classified from the Ebel standard setting. We also identified the relative strengths and weaknesses for each AKT profile group based on their average scores in each ICU. Similarly, we conducted the equivalent analyses with student profiles identified from OSCE data.

#### The relationship between AKT and OSCE profiles

To examine the extent to which AKT and OSCE profiles aligned with each other, we related the AKT data with the OSCE data. To be specific, we coded the profiles ordinally as 1, 2, 3,… according to the overall AKT/OSCE performance levels of the identified groups, and analysed the extent to which student AKT profiles predicted their OSCE profiles using ordinal regression modelling (Long & Freese, 2014).

## Results

### The AKT profiles

The results of LPA on the AKT data suggest the 4-profile model is the optimal solution, as it has the lowest AIC and BIC values (see Table 2, shaded row).

**Table 2:** LPA statistics of student performance in the AKT

Model	ll(model)	df	AIC	BIC	Entropy
2 – profile	-5376.98	16	10785.95	10844.94	0.80
3 – profile	-5332.65	22	10709.29	10790.4	0.75
4 – profile	-5308.28	28	10672.57	10775.8	0.73
5 – profile	-5305.67	34	10679.34	10804.7	0.67
6 – profile	-5298.53	40	10677.07	10824.55	0.76

In this model, the entropy value 0.73 is also acceptable according to the usual guidelines. The average posterior probabilities of the classification of the profile membership range from 0.82 to 0.97, which are all above the recommended cut-off of 0.80. Four distinct student profiles corresponding to the characteristics related to their achievements in the five specialities in AKT were therefore identified. The four profiles AKTgroup1, AKTgroup2, AKTgroup3, and AKTgroup4 share 3.4%, 23.7%, 41.4% and 31.5% respectively of the total student sample (see Table 3).

The student groups identified based on the four-profile solution also generally resonate with the pass/fail^2^ classification of students based on Ebel standard setting. As Table 3 shows, all AKTgroup1 students clearly failed the AKT Sequence 1 exam; all the AKTgroup3 and AKTgroup4 students are those clearly passed the exam; and AKTgroup2 includes the group of students whose performance was relatively close to the borderline. This data further supports the meaningful interpretation of the four-profile solution.

**Table 3:** Statistics of the groups in the 4-profile solution

AKT Profile	Sample *n* (%)			Average probabilities for most likely latent profile membership			
	Pass	Fail	Total	1	2	3	4
AKTgroup1	0 (0.00%)	10 (3.39%)	10 (3.39%)	**0.97**	0.00	0.00	0.03
AKTgroup2	41 (13.90%)	29 (9.83%)	70 (23.73%)	0.01	**0.86**	0.13	0.00
AKTgroup3	122 (41.36%)	0 (0.00%)	122 (41.36%)	0.00	0.11	**0.82**	0.07
AKTgroup4	93 (31.53%)	0 (0.00%)	93 (31.53%)	0.00	0.00	0.17	**0.83**

The mean achievements of these four groups of students in the five specialities in the AKT are displayed in Table 4. One-way ANOVA post hoc tests suggest that the between-group differences in the five specialities are mostly statistically significant with moderate to large effect sizes (*r*^*2*^ ranged from 0.40 to 0.60), except for the difference between AKTgroup1 and AKTgroup2 in ACC (*p* = 0.2).

**Table 4:** Mean differences between the AKT profiles in the specialities

Speciality	AKTgroup1 M (SD)	AKTgroup2 M (SD)	AKTgroup3 M (SD)	AKTgroup4 M (SD)	F (3, 291)	*p*	*r*^2^
ACC	43.67 (10.71)	49.62 (8.93)	59.26 (8.00)	71.29 (7.72)	108.52	< 0.001	0.53
CCC	41.33 (10.21)	54.76 (7.69)	61.12 (6.70)	68.96 (6.27)	85.71	< 0.001	0.47
GOSH	52.50 (5.34)	64.44 (6.30)	75.00 (6.48)	81.22 (6.62)	127.97	< 0.001	0.57
Paediatrics	41.33 (8.92)	54.29 (7.7)	64.95 (6.61)	73.55 (6.31)	143.06	< 0.001	0.60
Psychiatry	47.59 (12.36)	66.45 (8.96)	72.87 (8.13)	79.09 (6.63)	65.64	< 0.001	0.40

**Fig. 1:**
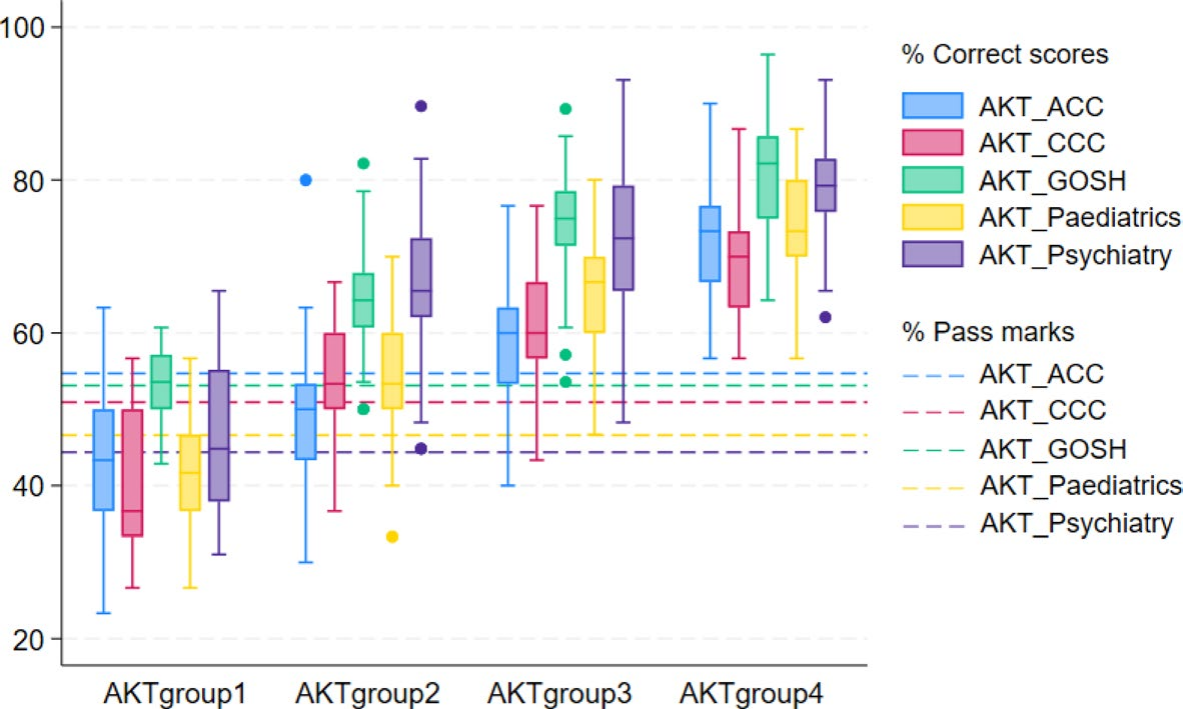
Distribution of the AKT performance of the four-profile solution

Figure 1 graphically illustrates the distribution of the speciality-level AKT performance of the four groups. On average, the lowest achievers AKTgroup1 consistently failed to correctly answer half of the items on four of the specialities, and almost all of them failed to achieve the specialty-level ‘pass marks’ on the ACC, CCC, and Paediatrics items. AKTgroup2 had more mixed average scores across the specialities, doing well in GOSH and Psychiatry but failing to pass ACC. AKTgroup3, compared with AKTgroup1 and AKTgroup2, had higher average scores; However, some students in this group obtained a score lower than 60% or even 50% in some of the speciality areas (especially ACC and CCC). For the highest-achiever group AKTgroup4, all the students achieved well in all the specialities.

In summary, we conclude that the LPA using the AKT outcomes has worked well based on the usual diagnostics/model fit criteria (e.g. Akaike, 1974; Muthén, 2004; Schwarz, 1978; Weller et al., 2020), producing four groups of students with successively improving performance across the five specialties.

### The OSCE profiles

As to LPA results of students’ OSCE data, Table 5 shows that BIC values are similar in the 2-profile and 3-profile models and increased in the other models. AIC values dropped dramatically from 2-profile model to 3-profile model. These data suggest that the 3-profile model is preferred (shaded). This model also has the entropy value (0.82) much higher than that in other models. Moreover, it also had acceptable average posterior probabilities, which range from 0.84 to 0.92 (see Table 6).

**Table 5:** LPA statistics of student performance in the OSCE ICU stations

Model	ll(model)	df	AIC	BIC	Entropy
2 – profile	-6767.96	19	13573.92	13643.98	0.72
3 – profile	-6748.8	26	13549.6	13645.46	0.82
4 – profile	-6739.71	33	13545.42	13667.09	0.67
5 – profile	-6734.06	40	13548.12	13695.6	0.66
6 – profile	-6731.89	47	13557.79	13731.08	0.66

From Table 6, we can see that OSCEgroup1 students all failed the OSCE (standard setting based on borderline regression); about 55% of students grouped into OSCEgroup2 passed the OSCE; and all OSCEgroup3 students clearly passed the OSCE. The three profiles OSCEgroup1, OSCEgroup2, and OSCEgroup3 made up 1.7%, 46.1%, and 52.2% of the student sample respectively.

**Table 6:** Statistics of the groups in the 3-profile solution

OSCE Profile	Sample *n* (%)			Average probabilities for most likely latent profile membership		
	Pass	Fail	Total	1	2	3
OSCEgroup1	0 (0.00%)	5 (1.69%)	5 (1.69%)	**0.84**	0.16	0.00
OSCEgroup2	75 (25.42%)	61 (20.68%)	136 (46.10%)	0.00	**0.91**	0.09
OSCEgroup3	154 (52.20%)	0 (0.00%)	154 (52.20%)	0.00	0.08	**0.92**

As shown in Table 7, students in OSCEgroup1 had the lowest mean performance across the specialities in the OSCE; OSCEgroup2 was the group of moderate performers; and OSCEgroup3 had the highest levels of performance. According to one-way ANOVA post hoc tests, the overall between-group differences are statistically significant with small or moderate effect sizes (*r*^*2*^ ranged from 0.23 to 0.44), except for the differences between OSCEgroup 1 and OSCEgroup2 in Paediatrics stations and in ICU-integrated stations (*p*_*Paediatrics*_ = 0.8, *p*_*ICUint*_ = 0.2).

**Table 7:** Mean differences between the OSCE profiles in the specialities

Speciality	OSCEgroup1 M (SD)	OSCEgroup2 M (SD)	OSCEgroup3 M (SD)	F (2, 292)	*p*	*r*^2^
ACC	25.71 (6.51)	42.36 (11.13)	56.26 (11.88)	63.73	< 0.001	0.30
CCC	28.21 (8.22)	54.84 (9.39)	66.76 (8.30)	100.21	< 0.001	0.41
GOSH	37.86 (10.96)	54.66 (8.67)	68.39 (8.11)	116.25	< 0.001	0.44
Paediatrics	31.27 (14.17)	37.94 (12.06)	52.21 (14.04)	45.31	< 0.001	0.24
Psychiatry	47.93 (11.07)	59.14 (10.08)	69.42 (9.95)	44.81	< 0.001	0.23
Integrated	51.89 (9.71)	58.63 (8.36)	68.00 (7.89)	52.97	< 0.001	0.27

**Fig. 2:**
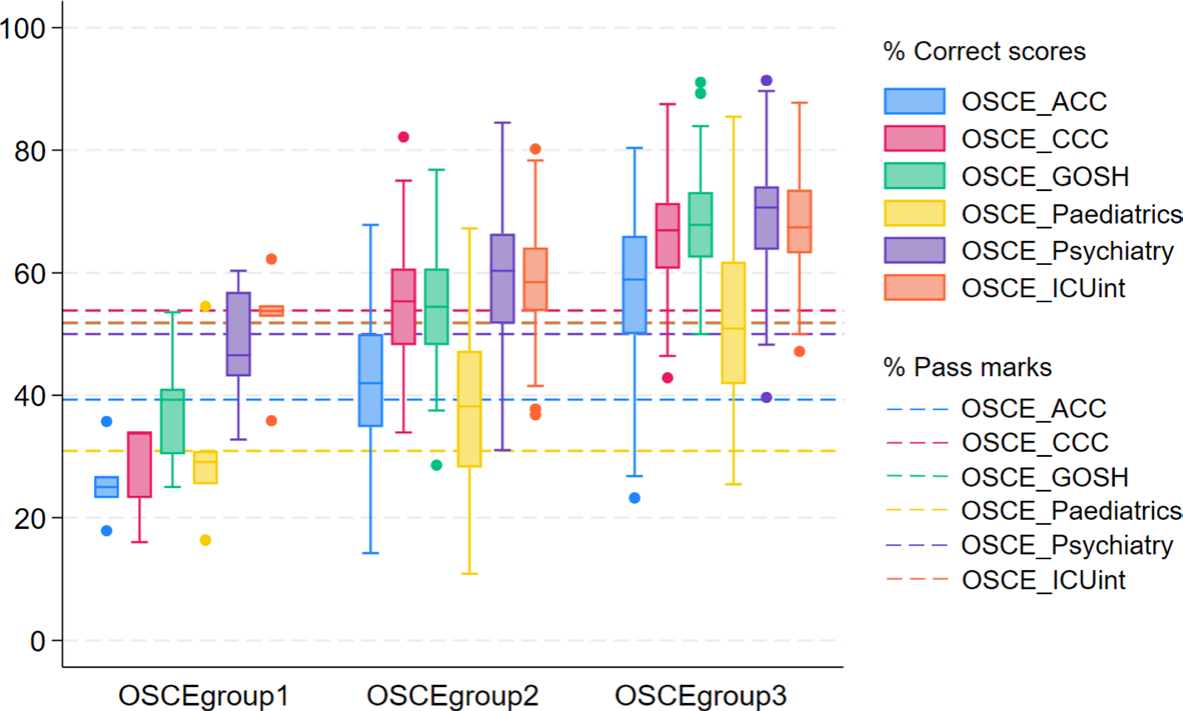
Distribution of OSCE performance of the three-profile solution

Figure 2 displays the OSCE performance distribution across the six predictor variables for the three groups. In general, OSCEgroup 1 had scores lower than 40% in the stations of ACC, CCC, GOSH and Paediatrics and almost all students in this group failed these stations. However, they mostly passed the ICU integrated stations. Compared with OSCEgroup1, OSCEgroup2 had higher and more consistent performance across CCC, GOSH, Psychiatry and integrated stations. Most students in this group passed Psychiatry and integrated stations. Their relatively low performance in other stations meant that 45% of this group failed the OSCE. For OSCEgroup3, students generally performed well across the stations, although had relatively low scores in ACC and Paediatrics.

In summary, the LPA using the OSCE outcomes has also worked well based on the usual diagnostics/model fit criteria, this time producing three groups of students with successively improving performance across the specialties and integrated stations.

### The relationships between AKT and OSCE profiles

Comparing the AKT and OSCE profiles, we find that they aligned with each other to an extent (Spearman’s rho = 0.45, *p* < 0.001, a moderate, but statistically significant, association). Through running an ordinal regression model, we found that the AKT profile variable significantly and positively predicted OSCE profiles (*p* < 0.001, pseudo R^2^ = 0.15).

As shown in Table 8, a crosstab of the distribution of students’ AKT and OSCE profiles, most of AKTgroup1 and AKTgroup2 students belonged to OSCEgroup1 and OSCEgroup2. Most of AKTgroup3 and AKTgroup4 students were in OSCEgroup3, and no students in these two high AKT profiles were in OSCEgroup1. However, it is noteworthy that there were some AKTgroup1 and AKTgroup2 students demonstrating very good skills in OSCE (i.e. grouped into OSCEgroup3). Also, a number of AKTgroup4 students were in OSCEgroup2, indicating that they did not perform as well in the OSCE as they did in the AKT.

**Table 8:** Cross tabulation of students’ AKT and OSCE profiles

AKT profiles	OSCE profiles			
	OSCEgroup1	OSCEgroup2	OSCEgroup3	Total
AKTgroup1	2	7	1	10
AKTgroup2	3	51	16	70
AKTgroup3	0	58	64	122
AKTgroup4	0	20	73	93
Total	5	136	154	295

## Discussion

Using latent profile analysis of summative assessment data, this study identified four distinct groups of students according to performance patterns in the AKT, and three such groups in the OSCE. Such identification is more fine-grained than single-score based pass/fail classifications, in terms of improving feedback for tailored remediation purpose. According to the usual methodological guidelines (Nylund et al., 2018; Spurk et al., 2020; Weller et al., 2020), the LPA worked well suggesting that these distinct groupings provide meaningful and substantive information about the performance of sub-cohorts of students. For each identified group, we also investigated relative strengths and weaknesses across the five specialities used as indicators in the analysis. The relationship between students’ AKT profiles and their OSCE profiles generally showed a positive correlation between the two performances, but did reveal some important variation from this for some individual students.

### Areas for feedback and development

In terms of the practical use of the findings, these classifications can be used to provide more meaningful and effective feedback and to highlight to faculty the specific areas that the lower performing groups need additional support for where appropriate at their later learning phase (e.g. Year 4 resit, or Year 5). The AKT results identify specific learning needs for all four student groups, but particularly for the two lower performing ones who need remediation. For clinical tutors and placement supervisors, the analysis can be used to help them to work collectively to support student learning with tailored strategies and placement management for different groups of students. For example, this include providing AKTgroup1 with additional learning support across all the speciality areas, but especially in ACC, CCC, and Paediatrics, and providing more support for AKTgroup2 in ACC (most of whom were close to or below borderline in this speciality).

There is also, perhaps, some important messages in the analysis for elements of the teaching and curriculum – students appear to not have performed as well, relatively, in ACC, compared to the other specialties. This finding supports extant studies which argue that acute care is perceived to be a concerned area for improvement in medical student learning (Burridge et al., 2020; Tallentire et al., 2012). According to the year 4 curriculum at School of Medicine at Leeds, amongst the five specialities, ACC is skill-intensive and also the only one incorporating four sub-specialities (i.e. acute medicine, emergency medicine, critical care and anaesthesia), which suggests that it might be more demanding and challenging for students. Future curriculum reviews of ACC might consider wider issues of training, student absence, professionalism and so on help ensure students’ sufficient exposure to all the requisite skills, in order to improve performance in ACC (Burridge et al., 2020; Burford et al., 2014). As displayed in Table 1; Fig. 1, students, regardless of their AKT profiles, had relatively low scores in ACC and CCC, and high scores in Psychiatry; however, the pass marks set based on Ebel ratings for these ACC and CCC were relatively high, and for Psychiatry was lowest. These data can be used to inform reviews of teaching, item difficulties and the Ebel standard setting.

Moving on to the OSCE findings, the performance patterns also inform clinical tutors and placement supervisors on delivering adaptive clinical teaching and providing tailored support for the two distinct subgroups identified as in need of particular remediation. OSCEgroup1 particularly need very substantial support (e.g. learning opportunities, supervision, feedback, etc.) in ACC, CCC, GOSH, Paediatrics and Psychiatry as they generally achieved much lower than the pass marks in most of the related stations, while OSCEgroup2 could benefit from additional support in ACC, CCC, GOSH, Paediatrics to achieve clear passes in these domains.

Students in all three OSCE profiles generally obtained lower scores in ACC and Paediatrics. This generally aligned with their AKT performance patterns in terms of the relative weakness in ACC. Regarding the relatively poor clinical performance in Paediatrics, O’Donoghue et al. (2018) makes the point that students generally have limited exposure to cases in this speciality compared to adult medicine in undergraduate studies. As with the AKT, these findings can also be used to inform reviews of teaching and station development.

It is noteworthy that even the weakest performers (OSCEgroup1) did relatively well in the integrated stations (Fig. 2). That all sub-groups of students did well on these integrated stations might have wider implications for their validity compared to those stations that are specialty specific and therefore inform curriculum development. The testing context where a specific case scenario is focused in a station may sometimes inhibit students from mobilising their wider knowledge and skills to, for example, comprehensively collect and interpret information from patients (Blaskiewicz et al., 2004).

Our study found a clear overall relationship between AKT and OSCE classifications, but also adds to the evidence suggesting that there can be inconsistency between students’ performance in these two assessment formats (Nguyen et al., 2023). In particular, we found some low AKT achievers did well in the OSCE while some high AKT achievers did not do consistently well in the OSCE. As is well known, a student’s limited clinical knowledge and skills can, to some extent, be compensated by relatively good communication skills or other generic skills to achieve a good overall score in OSCE (Boscardin, 2012; Setyonugroho et al., 2015). Conversely, a student’s OSCE performance may be negatively influenced if they have, for example, a deficit in communication skills, despite having good knowledge skills. According to Attrill et al. (2016) and Groene et al. (2022), students’ communication skills can be moderated by their demographics such as gender and native language background. Also, examiners, depending on their experiences, stringency, etc., can also have important effects on students’ OSCE performance (Chong et al., 2017; Homer, 2023).

### Pass/fail decision-making

Our work shows that some students in some groups in both the AKT and OSCE did not meet the minimum requirements for some specialities. This is especially the case for the mixed groups which include students who have met the required standard to pass overall, and those who have not (i.e. AKTgroup2 and OSCEgroup2). This finding raises the question of our conception of borderline students and the approach to defining and identifying them (Homer et al., 2017). Sequential models of assessment are more likely to correctly classify such students through seeking more information on weaker/borderline candidates before final pass/fail decisions are made (Cookson et al., 2011; Muijtjens et al., 2000; Pell et al., 2013). To meet the curriculum requirements for fully preparing students to be qualified doctors (GMC, 2015), Boscardin (2012) argues that cut-off scores for each clinical domain also need to be involved in pass/fail decision making. However, this raises issues around reliability of sub-domains (Sinharay, 2010), and is likely to increase failure rates.

### Study limitations and future research

Our results of the profile classification in this study were based on the data of one Year 4 cohort in one medical school, and therefore may not be generalisable for other cohorts or other school contexts. However, the approach of profiling students’ performance based on summative assessment data as demonstrated in our study, and our findings, could provide models for research focusing on other cohorts or students in different year groups in other institutions, and for research aimed at generating more informative feedback from summative assessment data. If future replication studies with the data of different cohorts were to find similar results (i.e. consistent patterns of relative weaknesses and strengths), this would help confirm specific areas of teaching, curriculum, or placements which need to be reviewed and, perhaps, improved. Finally, our analyses did not account for student demographics (e.g. gender, native language, learning disabilities, etc.), which might have some influence on their (in)consistent performance between the AKT and OSCE. These background factors can be considered in future research investigating and comparing students’ performance across assessment formats.

## Data Availability

No datasets were generated or analysed during the current study.

## Footnotes

1 Latent profile analysis is based on data of continuous variables, while latent class analysis employs categorical variables.

2 The “pass/fail” here and in what follows refers to the classification of students who have passed the part 1 of the sequential AKT/OSCE or not. Students who have not passed part 1 will have to take part 2.
